# Lanthanide Content and Adulteration Testing of Greek Honeydew and Nectar Honey

**DOI:** 10.3390/molecules31152685

**Published:** 2026-08-01

**Authors:** Eleni C. Mazarakioti, Vassilios K. Karabagias, Sofia Karabournioti, Dimitrios G. Lazaridis, Anastasios Zotos, Angelos Patakas, Athanasios Ladavos, Ioannis K. Karabagias

**Affiliations:** 1 Department of Food Science & Technology, School of Agricultural Sciences, University of Patras, G. Seferi 2, 30100 Agrinio, Greece; e.mazarakioti@ac.upatras.gr (E.C.M.); vkarampagias@upatras.gr (V.K.K.); dlazaridis@ac.upatras.gr (D.G.L.); apatakas@upatras.gr (A.P.); alantavo@upatras.gr (A.L.); 2 Attiki Bee Culturing Co.—Alex. Pittas S.A., 9 Protomagias Street, Kryoneri, 14568 Athens, Greece; skar@attiki-pittas.gr; 3 Department of Sustainable Agriculture, University of Patras, G. Seferi 2, 30100 Agrinio, Greece; azotos@upatras.gr

**Keywords:** honey, lanthanides, sugar addition, botanical origin, chemometrics

## Abstract

Given the scarce data in the literature regarding the lanthanide content of Greek monofloral honey, Sc (Scandium), Y (Yttrium), La (Lanthanum), Ce (Cerium), Pr (Praseodymium), Nd (Neodymium), Sm (Samarium), Eu (Europium), Gd (Gadolinium), Tb (Terbium), Dy (Dysprosium), Ho (Holmium), Er (Erbium), Tm (Thulium), Yb (Ytterbium), Lu (Lutetium) and Th (Thorium) were determined in honeydew and nectar honey, using inductively coupled plasma mass spectrometry (ICP-MS). Results showed that, in general, honeydew honey had a higher lanthanide content than nectar honey, even though the determined values were below the limit of quantification. Preliminary reference values in this case for pine honey were Ce (26.82 ± 21.49 ng/L) and La (15.25 ± 11.82 ng/L). Adulteration testing was also performed using elemental analysis coupled to isotope ratio mass spectrometry (EA-IRMS), where no adulteration with cheap sugars was detected. Factor analysis identified the principal lanthanides related to honey botanical origin, basically Ce, and the *k*-NN algorithm classified the samples, achieving a training classification rate of 81.8% and a hold-out classification rate of 76.5%. Even in low amounts, lanthanides may contribute to the botanical origin identification of monofloral honey.

## 1. Introduction

Council Directive 2001/110/EC [1] defines honey as the natural sweet substance produced by *Apis mellifera* honeybees. It comes from the nectar of flowers or from the honeydew secretions of scale insects on conifer trees that honeybees collect. Honey consists essentially of different sugars, predominantly fructose and glucose (approx. 77% to 78%), water (approx. 14% to 18%), and numerous microconstituents including organic acids, amino acids, proteins, enzymes, minerals and trace elements, vitamins, phytochemicals, as well as solid particles originating from the collection of honey [2]. Council Directive 2001/110/EC [1] supports the natural character of honey and prohibits any human intervention that could affect the composition of honey, such as the addition of foreign components or food additives.

The sensory characteristics of honey, including color, aroma, and taste, are affected by the types of flowers from which the pollen is collected, the geographical region of collection, and the climate of this area. In addition, the type of bees used for honey production (honeybees or stingless bees) may affect the compositional and sensorial characteristics of honey [3].

The last years, the primary focus of the food industry and honey suppliers has been to develop protocols for authenticating honey. Speaking of authentication, the term refers to the accurate determination of the botanical and geographical origin of honey based on its distinctive pollen characteristics, physicochemical parameters, and sensory or nutritional/biological properties. Among the official methods to determine the botanical origin of honey is melissopalynology. This method examines the morphology of pollen counts in each honey sample under a microscope [4]. Even though this is the official method for the botanical characterization of honey, it has often been characterized as a method requiring much time for analysis and experts to evaluate the results accurately. In addition, in some cases, different analysts [5] cannot robustly adopt it, as in the case of under-represented pollen or discrepancies during the analysis. Taking advantage of these observations, the research community is trying to seek alternative methodologies to authenticate honey. Conventional physicochemical parameters (i.e., moisture, sugars, electrical conductivity, free acidity, pH, ash, etc.) [6] and sensory characteristics (i.e., color, aroma, taste) [7], gas and liquid chromatography or nuclear magnetic resonance or infrared spectroscopy [8], laser-induced breakdown spectroscopy [9], and DNA-based methods [10] alone or in combination, have been used in the recent years in conjunction with chemometric techniques [11] to indicate the specific physicochemical markers such as volatile compounds [12], phenolic compounds [13,14], minerals and trace elements [15] or carbon isotope ratios [16]. In this context, we should not forget that microconstituents of honey can provide useful information regarding its geographical and botanical origin [17].

Speaking of minerals, among those parameters of honey authentication, the lanthanides are a group of metallic elements that includes at least 14 elements, with atomic numbers ranging from 57 to 70, from lanthanum through ytterbium. However, some rare earth elements, such as scandium (atomic number 21) and yttrium (atomic number 39), are also included in this group [18] under broader usage, not the strict IUPAC definition. Regarding their presence in Greek honeydew or nectar honey, no previously published article in the literature reports their content. In a recent study, the lanthanide content of Italian honey of different botanical origins (acacia, chestnut, clover, linden, and taraxacum) and geographical origins (Turin, Asti, Cuneo, and Vercelli) was considered as a potential parameter of the geographical and botanical origin of nectar or honeydew honey, indicating probable different bioaccumulation of lanthanides in nectar plants or trees grown in a specific region with a distinctive geological substrate and mineralogical composition [19]. In addition, numerous studies have highlighted that specific lanthanides such as cerium, lanthanum, and gadolinium alone [20,21] or in combination with other biologically active molecules such as coumarin [21,22,23] can serve as anticancer agents.

On the other hand, for the quality control analysis of honey, the detection of added cheap sugars (i.e., corn or cane sugar) in honey or honeybees feeding with these sugars, elemental analysis–isotope ratio mass spectrometry (EA-IRMS) or in combination with liquid chromatography (EA/LC-IRMS) comprises the most accurate analytical method to detect such adulteration [24,25], even though conventional and real-time polymerase chain reaction (PCR) have also been used for the same purpose [26]. Given that C4 plants assimilate and stabilize more carbon dioxide from the environment than C3 plants, the proportion of carbon-13 to carbon-12 (^13^C/^12^C) of these plants is higher than that of C3 plants. This ratio/value in C4 plants is around −8‰ to −16‰, but this number in C3 plants is identical to −22‰ to −32‰. Bees ordinarily feed on blooming plants of the C3 pathway. Subsequently, the ^13^C/^12^C in nectar is indicative of C3 plants. As a result, if falsification is done utilizing other plants, including C4 plants, this could be detected by analyzing changes in the isotopic proportion [27].

Considering the abovementioned, the present study aimed to determine lanthanides in different types of Greek honeydew and nectar honey samples collected from 14 different regions in Greece, and test if any addition of cheap sugars had been done. An additional effort was carried out to classify samples according to botanical origin using the lanthanide content and chemometric techniques such as the *k*-nearest neighbors algorithm, and to find any correlations among lanthanide content and botanical origin of honey using factor analysis. To our knowledge, there is no previous study in the literature determining and reporting quantification results for lanthanide content in Greek fir, pine, and *Quercus ilex* honeys (honeydew honey), along with polyfloral/floral, citrus and thyme honeys (nectar honey), whereas only one study is available in the international literature [19] reporting data on lanthanide content of Italian honey and thus comprising the pure novelty of the present study.

## 2. Results and Discussion

### 2.1. Lanthanide Content of Greek Honeydew and Nectar Honey

Table 1 shows the lanthanide content (ng/L) in honey samples of different botanical origins and whether the data of each element falls within the limit of detection (LOD) or is below LOD (<LOD). In addition, different lowercase letters indicated statistically significant (*p* < 0.05) differences among honey samples of different botanical origin based on Tukey’s honestly significant difference (HSD) test. More specifically, Tm and Lu were not identified in any of the honey samples analyzed; therefore, these were excluded from Table 1 and statistical analysis. Pine honey samples had the highest average total lanthanide content (82.26 ng/L), followed by *Quercus ilex* (67.85 ng/L), fir (67.73 ng/L), and nectar (46.26 ng/L) honey samples. The most abundant lanthanides were Ce (26.82 ± 21.49 ng/L) and La (15.25 ± 11.82 ng/L) in pine honey samples. On the other hand, fir honey samples showed the lowest Tb content (0.04 ± 0.14 ng/L) among honeydew honey samples, whereas Tb and Ho were not identified in nectar honey samples. In our case, only Ce showed significant (*p* < 0.05) between-group differences according to Tukey’s HSD test. In a previous study [19] regarding Italian acacia, chestnut, clover, linden, and taraxacum honey samples, the lanthanide content, even in small amounts as in the present study, proved to be characteristic of the geographical and botanical origin of honey. The most abundant lanthanide was Ce in chestnut honey, in agreement with the results of the present study. In addition, the authors [19] concluded that the mineral composition of soil, described among others by the lanthanide distribution, is transmitted unaltered from soil to flowers, and then to honey.

To the best of our knowledge, there is no literature available concerning the lanthanide content in honey, so further comparisons of the results cannot be done. What is evidenced by the results of Table 1 and those of the only study in the literature [19] concerning the fluctuations in lanthanide content is that both the geographical and botanical origin of honey may affect the lanthanide content. Finally, we believe that lanthanides could assist in the authentication of honey, given that they may be associated with the honeybees’ nectar or honeydew collection from a specific region and, in parallel, provide information on how their accumulation proceeds in plants or trees growing in this specific region (regional bioaccumulation). This hypothesis will be further supported by the analysis of soil samples of the regions of interest. In our case, the honey samples were collected from 14 different regions known to produce honeydew or nectar honey. In this context, we should not forget that, in general, honeydew honey has a higher mineral content compared to nectar honey [15,16], and as evidenced in the present study, lanthanides showed a similar pattern, as they were identified in higher content in honeydew honey samples compared to nectar ones.

### 2.2. Adulteration Testing

During EA-IRMS analysis, the expanded relative measurement uncertainty was 3% (coverage factor k = 2.58; confidence interval 99%) without taking the sampling into account. The values determined correspond to the QSI criteria for authentic honeys [24,28] and do not indicate the addition of foreign sugars [1]. The differences in protein-honey (P-H) according to the AOAC 998.12 method [28] were *δ*-^13^C ‰ = +0.08. In addition, no adulteration with C4-sugars was monitored (C4-sugar-content = 0.00) related to the average *δ*-^13^C value of corn syrup of −9.7 ‰ vs. V-PDB (Vienna Pee Dee Belemnite) standard (*δ*-^13^C value ~ 0.00). According to the AOAC 998.12 method [28], the target value to identify adulteration of honey with foreign sugars is ≤7.00%.

### 2.3. Lanthanides’ Contribution to the Botanical Origin Identification of Greek Honeydew and Nectar Honey Using FA and *k*-NN

#### 2.3.1. FA

**Figure 1:**
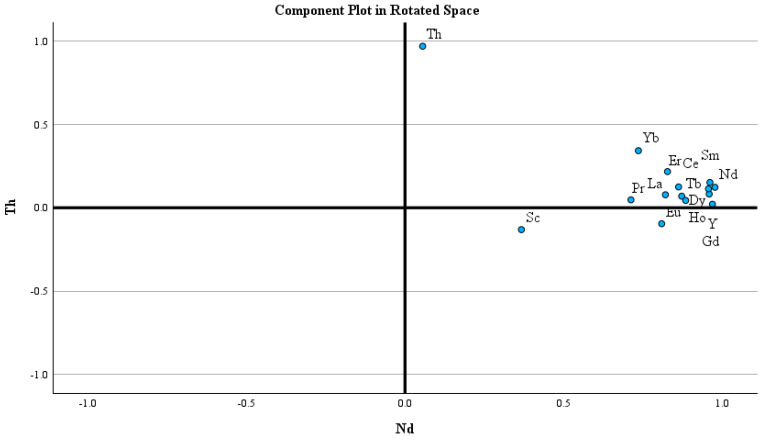
Lanthanides in the rotated space of factor analysis as correlating factors of the botanical origin of Greek honeydew and nectar honey.

To perform FA, metrics such as the Kaiser–Meyer–Olkin (KMO) index, which assesses sample adequacy (should be >0.50), and Bartlett’s Test of Sphericity, which assesses whether the correlations between the variables allow the application of factor analysis (should have a *p*-value *<* 0.05), were considered. Based on the above, the KMO index was 0.892, whereas Bartlett’s Test of Sphericity had the following values: Chi-Square (X^2^) = 1122.860, df = 105, *p* < 0.001, indicating the correct application of factor analysis and the presence of correlations among lanthanides with the botanical origin of honey. The principal components (PCs) that showed the highest correlation were two. Based on these PCs, the variance explained was 75.81%, which is considered satisfactory. The lanthanides for which the correlation values in the rotated component matrix of the multidimensional space of factor analysis were the largest were Nd (PC1, correlation of 0.978 and explained variance of 67.69%) and Th (PC2, correlation of 0.969 and explained variance of 8.12%) (Figure 1).

#### 2.3.2. *k*-NN

During the case processing summary of *k*-NN analysis, all cases, representing the number of samples (N = 50) and the measured parameters, were valid (100%). Then the initial sample (N = 50) was divided randomly based on the operation of *k*-NN into training (N = 33 samples, 78% of the total sample size) and holdout (N = 11 samples, 22% of the total sample size) partitions to estimate the classification ability of honey samples according to botanical origin based on lanthanide content. The *k*-NN algorithm divides the original dataset into training and holdout samples. The training sample includes the data used to train the nearest neighbor model. The holdout sample is an independent set of data that is used to assess the final model’s applicability. The error performance of the holdout sample gives the correct predictive ability of the model, as the holdout cases are not used to build the model [29]. During the analysis, the percentage of error in the training data set was 18.2%, and in the holdout data set was 23.5%. Table 2 shows the overall performance of the *k*-NN model in relation to the classification of honey according to botanical origin based on the determined lanthanides. The overall classification rate for the training data set was 81.8.%, and that of the holdout data set was 76.5%. Both are considered satisfactory, even though the limited samples of *Quercus ilex* honey may have a strong impact on the statistical power of Tukey’s HSD test.

## 3. Materials and Methods

### 3.1. Honey Samples

The samples that were possible to collect and introduced in the analysis were fifty monofloral honey samples [fir (13 samples), *Quercus ilex* (4 samples), nectar [13 samples consisting of polyfloral/floral (10 samples), thyme (2 samples), and orange (1 sample)], and pine (20 samples)]. Honey samples were collected from different regions in Greece (Parnonas, Argos, Evritania, Thassos, Thessaly, Karditsa, Evia, Pavliani, Evros, mountainous Nafpaktos, Kos, Kopaida, Halkidiki, Pilio) during the 2022 and 2023 harvesting periods from selected beekeepers. The botanical origin of honey samples was confirmed in the laboratory of Attiki Bee Culturing Co., Alex Pittas S.A., based on physicochemical [6] and sensory analyses [7] following an internal protocol. Samples were stored in plastic containers in a dark place at room temperature (20 ± 1 °C) and analyzed immediately upon shipment to the laboratory.

### 3.2. Determination of Lanthanides

The study focused on the determination of seventeen lanthanides, including Sc, Y, La, Ce, Pr, Nd, Sm, Eu, Gd, Tb, Dy, Ho, Er, Tm, Yb, Lu, and Th.

### 3.3. Chemicals, Calibration Standards, and Quality Control for Lanthanide Content Analysis (ICP-MS)

For the calibration of the instrument, multi-element standard solutions with certification were utilized, which were provided by Agilent Technologies (Santa Clara, CA, USA). Also, a Rh internal standard solution (10 µg/mL in 2% HCl) and an instrument-tuning solution with Li, Y, Ce, Tl, and Co (10 µg/mL in HNO_3_) were provided by the same producer. Working calibration solutions of the required concentration were made freshly at the levels of 0.0 (blank), 0.5, 1.0, 5.0, 10, and 20 µg/L to get the respective points in calibration curves for each analyte. The method sensitivity was evaluated through the determination of the limits of detection (LOD) and quantification (LOQ), calculated as 3 and 10 times the standard deviation of twelve procedural blanks, respectively. The corresponding values for each element are shown in Table 3. Values below the LOD/LOQ were treated as is for the statistical tests. Honeywell Fluka (Seelze, Germany) supplied high-purity nitric acid (70% *v*/*v*, TraceSELECT grade) and Sigma-Aldrich (Taufkirchen, Germany) provided 30% *w*/*w* hydrogen peroxide (ultra-trace analysis grade). Method accuracy was tested over the range of 80–120% of the certified values (Supplementary Table S1), indicating satisfactory analytical performance, using the IRMM-804 rice flour certified reference material (JRC-Geel, Geel, Belgium). The reference material was digested exactly as the honey samples. In Supplementary Figure S1, the calibration curves of all lanthanides are shown.

### 3.4. Instrumentation

An inductively coupled plasma mass spectrometer (Agilent Technologies 7850 series model, Santa Clara, CA, USA) coupled to a quadrupole analyzer was utilized for the elemental analysis of the seventeen target analytes (Sc, Y, La, Ce, Pr, Nd, Sm, Eu, Gd, Tb, Dy, Ho, Er, Tm, Yb, Lu, and Th). The Agilent 7850 model from Agilent Technologies, Santa Clara, CA, USA, was the specific mass spectrometer used for this study. Initially, an IntelliQuant approach [15] was employed to conduct a semi-quantitative screening for a wide variety of elements (up to 78 different elements across the periodic table); however, only those elements for which calibration standards were available were quantitatively assessed. The control of the instrument and the processing of the data were performed using MassHunter software version 5.1 (Agilent Technologies). A microwave-assisted digestion system was used to perform the digestion of the samples (Multiwave Go Plus, Anton Paar GmbH, Graz, Austria). A Smart2Pure water purification system (Thermo Electron LED GmbH, Langen-Selbold, Germany) was used to produce ultrapure water (18.2 MΩ·cm) suitable for trace element analysis. The operational conditions of the ICP-MS instrument are given in the Supplementary Materials (Supplementary Table S2).

### 3.5. Sample Preparation and Microwave Digestion

Teflon digestion vessels were used to weigh 500 mg of each honey sample accurately. The samples were treated with concentrated nitric acid (70%, 4 mL) and hydrogen peroxide (H_2_O_2_ 30%), 1 mL, and then subjected to microwave-assisted digestion with a two-step temperature program: ramping to 180 °C in 15 min and holding for 20 min at the same temperature. After the digestion, the vessels were cooled to room temperature. The resulting clear and slightly greenish solutions were transferred quantitatively to 50 mL polypropylene tubes, and then the volume was adjusted with ultrapure water. The samples prepared were kept in the dark at 4 °C until ICP-MS analysis. To avoid contamination, all the reusable laboratory items that were cleaned with diluted nitric acid soaking followed by rinsing with ultrapure water were used.

### 3.6. Adulteration Testing Using Elemental Analysis in Combination with Isotope Ratio Mass Spectrometry (EA-IRMS)

To detect such adulteration, elemental analysis coupled with isotope ratio mass spectrometry (EA-IRMS) as described in the AOAC 998.12 method [28] was used, taking into account weighing of the sample, sample preparation, and determination of the carbon isotopes for honey and protein. This approach can identify whether honey contains more than 7% of C-4 plant sugars, such as those derived from corn or cane sugar. The addition of C-4 sugars (cane sugar/corn syrup) to honey increases the value of the stable carbon isotope ratio (^13^C/^12^C) of honey. The ratio of the carbon isotopic value for protein (*δ*-^13^C ‰) in honey provides the standard to which the carbon isotope ratio value of the whole honey is compared. The difference between these values allows for the identification of the amount of the C-4 sugar content in honey and the estimation of its adulteration level. Both the honey and protein fraction must be analyzed on the same instrument [28]. The analysis was carried out in the certified laboratory Quality Services International (QSI) GmbH (Flughafendamm 9a, D-28199 Bremen, Germany).

### 3.7. Statistical Analysis

Data were subjected first to descriptive analysis to calculate the average and standard deviation values among samples of different botanical origins. For the application of multivariate statistics, the data related to lanthanide content were grouped by botanical origin (fir, pine, nectar, *Quercus ilex*), which served as the ordinal variable, and the lanthanide content data served as the scaled variables. Afterward, factor analysis (FA) was applied to find any correlations between lanthanides and the botanical origin of honey. FA is a dimension reduction technique and is categorized as an unsupervised statistical technique. FA provides the explained variance between the initial number of measured variables introduced in the analysis and a smaller number of extracted variables, called factors or principal components. The basic idea of FA is to summarize the relationships between the initial and the factor variables by concluding an explained total variance (% variance) related to those factors [15]. The Kaiser–Meyer–Olkin (KMO) examination serves as a statistical index for evaluating the appropriateness of the data for FA. This assessment evaluates the adequacy of sampling for individual variables in the model as well as for the overall model. The statistics represent the extent to which variance among the variables may be attributed to common variance. An increased proportion results in a higher KMO value, indicating greater suitability of the data for FA [30]. Principal component analysis (PCA) was used as the extraction method during FA, with the Varimax rotation and Kaiser Normalization [31].

For classifying samples by botanical origin based on lanthanide content, the k-nearest neighbors (*k*-NN) algorithm was used. K-NN is a common nonparametric method for classification and regression. It relies on distance metrics to find the nearest neighbor; these neighbors are then used for classification and regression tasks. To determine the nearest neighbor, Euclidean distance was applied [32]. To evaluate the classification performance of the algorithm, training and holdout partitions were considered during *k*-NN analysis. The number of neighbors used to build the model (selected predictors) was three (K = 3). Statistical analysis was performed using SPSS software v. 28.0 (SPSS, IBM Inc., Armonk, NY, USA).

## 4. Conclusions, Limitations, and Future Directives

The honeydew and nectar honey samples from Greece comply with the European standards regarding the absence of adulteration with cane or corn sugars, as evidenced by EA-IRMS. In addition, for the first time in the literature, data on the content of lanthanides in Greek honeydew and nectar honey using ICP-MS were reported. Even though lanthanides were determined in small amounts (ng/L), practically below the limit of quantification, the application of FA and *k*-NN analyses indicated the lanthanides that were most associated with the botanical origin of honey, and in parallel, classified honey samples according to botanical origin using training and holdout algorithms. In both cases, the overall variance explained exceeded 75%. However, we should stress that the limited *Quercus ilex* honey samples (owing to limited production) may comprise a limitation of the study regarding the classification performance of the *k*-NN model, so no clear findings, as in the other honey types tested, can be drawn. In addition, the lanthanide profiling at present has potential as a complementary tool rather than a definitive authentication method, given that there is no data on soil content and the complete bioaccumulation tests of lanthanides on the specific plants or conifer trees used for honey production. Nevertheless, the topic addressed in this study is new, and there is a need to conduct international research on the lanthanide content of honey from different botanical and geographical origins to more extensively study the factors that affect the ‘honey lanthanide fingerprint’ (HLF).

## Figures and Tables

**Table 1:** Lanthanide content (ng/L) of Greek honeydew and nectar honey.

Botanical Origin	Sc *	Y **	La **	Ce **	Pr *	Nd **	Sm **	Eu *	Gd **	Tb *	Dy **	Ho *	Er *	Yb *	Th **
Fir (N = 13)	3.65 ± 5.17 ^a^	10.00 ± 4.70 ^a^	10.00 ± 4.99 ^a^	18.35 ± 10.08 ^a^	2.27 ± 1.18 ^a^	7.73 ± 4.69 ^a^	2.11 ± 1.24 ^a^	0.46 ± 0.48 ^a^	1.61 ± 0.94 ^a^	0.04 ± 0.14 ^a^	1.46 ± 0.69 ^a^	0.08 ± 0.28 a	0.81 ± 0.43 ^a^	0.35 ± 0.43 ^a^	8.81 ± 19.86 ^a^
Nectar (N = 13)	7.96 ± 3.97 ^a^	6.54 ± 2.77 ^a^	7.69 ± 2.54 ^a^	11.92 ± 5.53 ^a^	2.38 ± 3.72 ^a^	4.19 ± 2.16 ^a^	1.27 ± 0.52 ^a^	0.15 ± 0.32 ^a^	0.85 ± 0.51 ^a^	0.00 ± 0.00 ^a^	0.88 ± 0.46 ^a^	0.00 ± 0.00 ^a^	0.31 ± 0.38 ^a^	0.31 ± 0.44 ^a^	1.81 ± 1.15 ^a^
Pine (N = 20)	6.72 ± 6.40 ^a^	10.50 ± 8.03 ^a^	15.25 ± 11.82 ^a^	26.82 ± 21.49 ^b^	2.52 ± 2.23 ^a^	9.00 ± 8.76 ^a^	2.27 ± 1.95 ^a^	0.55 ± 0.67 ^a^	1.70 ± 1.68 ^a^	0.15 ± 0.37 ^a^	1.62 ± 1.42 ^a^	0.15 ± 0.37 ^a^	0.67 ± 0.67 ^a^	0.42 ± 0.63 ^a^	3.92 ± 3.88 ^a^
*Quercus ilex* (N = 4)	0.62 ± 1.25 ^a^	9.12 ± 6.66 ^a^	10.50 ± 5.49 ^a^	20.25 ± 14.21 ^ab^	2.50 ± 2.04 ^a^	8.50 ± 8.03 ^a^	2.62 ± 1.93 ^a^	0.87 ± 0.25 ^a^	2.00 ± 2.00 ^a^	0.25 ± 0.50 ^a^	1.75 ± 1.85 ^a^	0.25 ± 0.50 ^a^	0.62 ± 0.63 ^a^	0.50 ± 0.41 ^a^	7.50 ± 10.68 ^a^

N: number of samples. * <LOD. ** <LOD and within LOD. Different letters in each column indicate statistically significant differences according to Tukey’s honestly significant difference (HSD) at the confidence level *p* < 0.05.

**Table 2:** Classification of honeydew and nectar honey according to botanical origin based on lanthanide content and *k*-NN.

Classification Table						
Partition	Observed	Predicted				
		Fir	Nectar	Pine	*Quercus ilex*	Percent Correct
Training	Fir	8	0	0	0	100.0%
	Nectar	0	9	1	0	90.0%
	Pine	2	1	10	0	76.9%
	*Quercus ilex*	0	0	2	0	0.0%
	Overall Percent	30.3%	30.3%	39.4%	0.0%	81.8%
Holdout	Fir	4	0	1	0	80.0%
	Nectar	0	2	1	0	66.7%
	Pine	2	0	5	0	71.4%
	*Quercus ilex*	0	0	0	2	100.0%
	Missing	0	0	0	0	
	Overall Percent	35.3%	11.8%	41.2%	11.8%	76.5%

**Table 3:** Limits of detection and quantification (ng/L) and linearity coefficients of determination (R^2^) of the calibration curves.

Element	LOD (ng/L)	LOQ (ng/L)	R^2^
**Sc**	22.54	75.13	1.0000
**Y**	4.27	14.22	0.9999
**La**	6.41	21.37	1.0000
**Ce**	12.65	42.18	1.0000
**Pr**	2.60	8.66	1.0000
**Nd**	3.49	11.65	0.9999
**Sm**	2.70	9.00	0.9999
**Eu**	2.66	8.88	1.0000
**Gd**	2.70	9.00	0.9999
**Tb**	1.95	6.51	1.0000
**Dy**	1.86	6.22	1.0000
**Ho**	2.70	9.00	1.0000
**Er**	1.95	6.51	1.0000
**Tm**	2.70	9.00	0.9999
**Yb**	2.70	9.00	0.9999
**Lu**	2.66	8.88	1.0000
**Th**	5.03	16.76	1.0000

LOD: limit of detection; LOQ: Limit of Quantitation; R^2^: represents the coefficient of linearity.

## Data Availability

The original contributions presented in this study are included in the article/Supplementary Materials. Further inquiries can be directed to the corresponding author.

## Supplementary Materials

The following supporting information can be downloaded at https://www.mdpi.com/article/10.3390/molecules31152685/s1, Table S1. Method of accuracy expressed as recovery (%) for metals in reference sample IRMM-804 Rice Flour; Table S2. ICP-MS operating parameters (agilent 7850); Figure S1. ICP-MS calibration performance for the investigated analytes/lanthanides.
