# Supplementary material for: Lanthanide Content and Adulteration Testing of Greek Honeydew and Nectar Honey
Source: Molecules. 2026 Aug 1;31(15):2685. doi: 10.3390/molecules31152685 (PMC13468225; doi:10.3390/molecules31152685)
Supplement: Supplementary file 1 [file molecules-31-02685-s001.zip › molecules-4390550-supplementary.pdf]

Supplementary Table S1. Method of accuracy expressed as recovery (%) for metals in reference sample IRMM-804 Rice Flour.

Element	Certified value (mg/kg)	Measured value (mg/kg)	%Recovery
**As**	0.049	0.047	96%
**Cd**	1.61	1.61	100%
**Cu**	2.74	2.96	108%
**Zn**	23.1	20.8	90%
**Mn**	34.2	35.9	105%
**Pb**	0.42	0.46	110%

Supplementary Table S2. ICP-MS operating parameters (Agilent 7850).

Parameter	Value
**RF Power**	1550 W
**Sampling Depth**	8.0 mm
**Nebulizer Gas**	1.05 L/min
**Helium Cell Gas**	4.3 mL/min
**Energy Discrimination**	3.0 V
**S/C Temperature**	2 ^{o}C

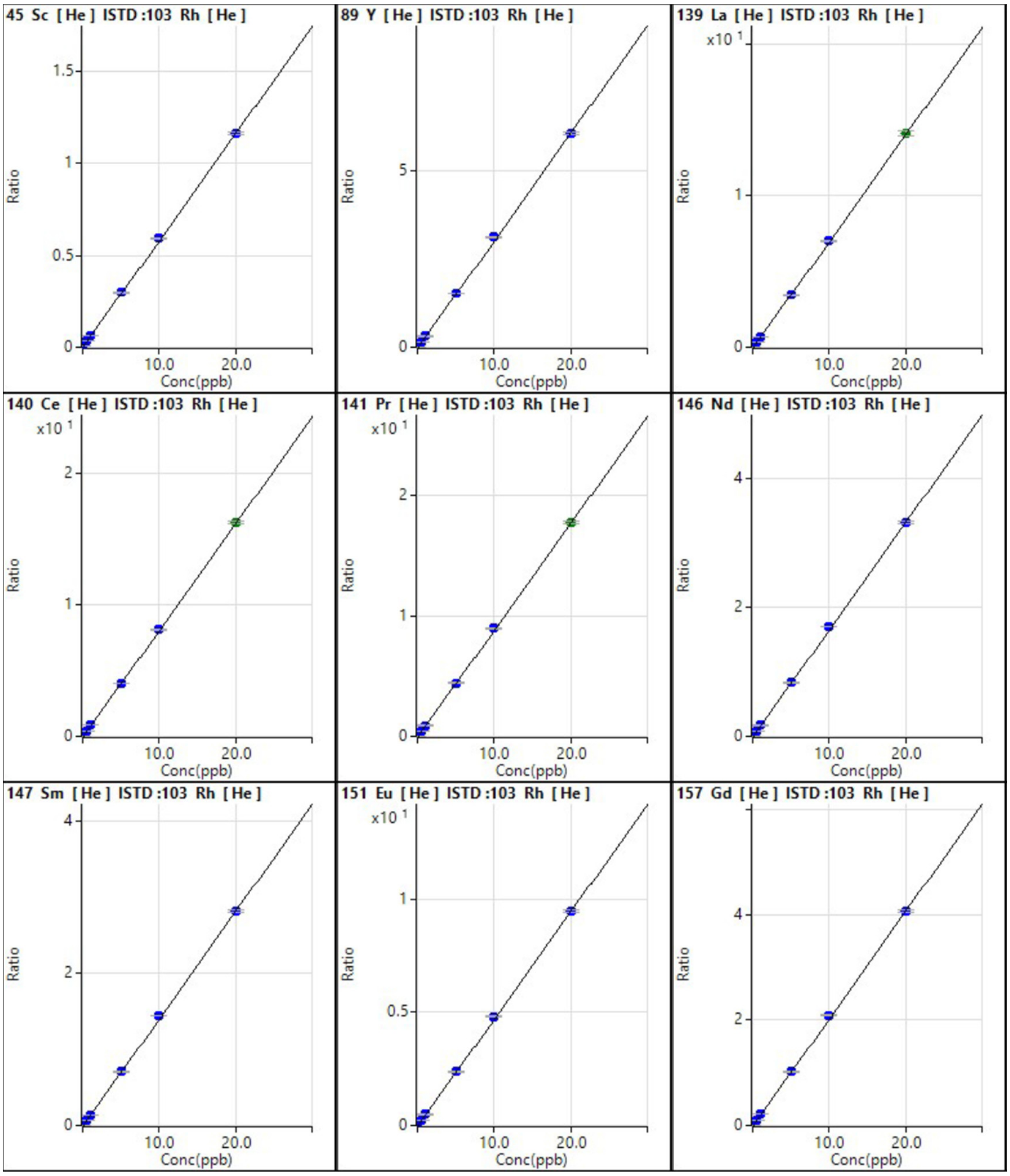
45 Sc [He] ISTD :103 Rh [He]
89 Y [He] ISTD :103 Rh [He]
139 La [He] ISTD :103 Rh [He]
140 Ce [He] ISTD :103 Rh [He]
141 Pr [He] ISTD :103 Rh [He]
146 Nd [He] ISTD :103 Rh [He]
147 Sm [He] ISTD :103 Rh [He]
151 Eu [He] ISTD :103 Rh [He]
157 Gd [He] ISTD :103 Rh [He]
Ratio
Conc(ppb)
x10 1
10.0
20.0

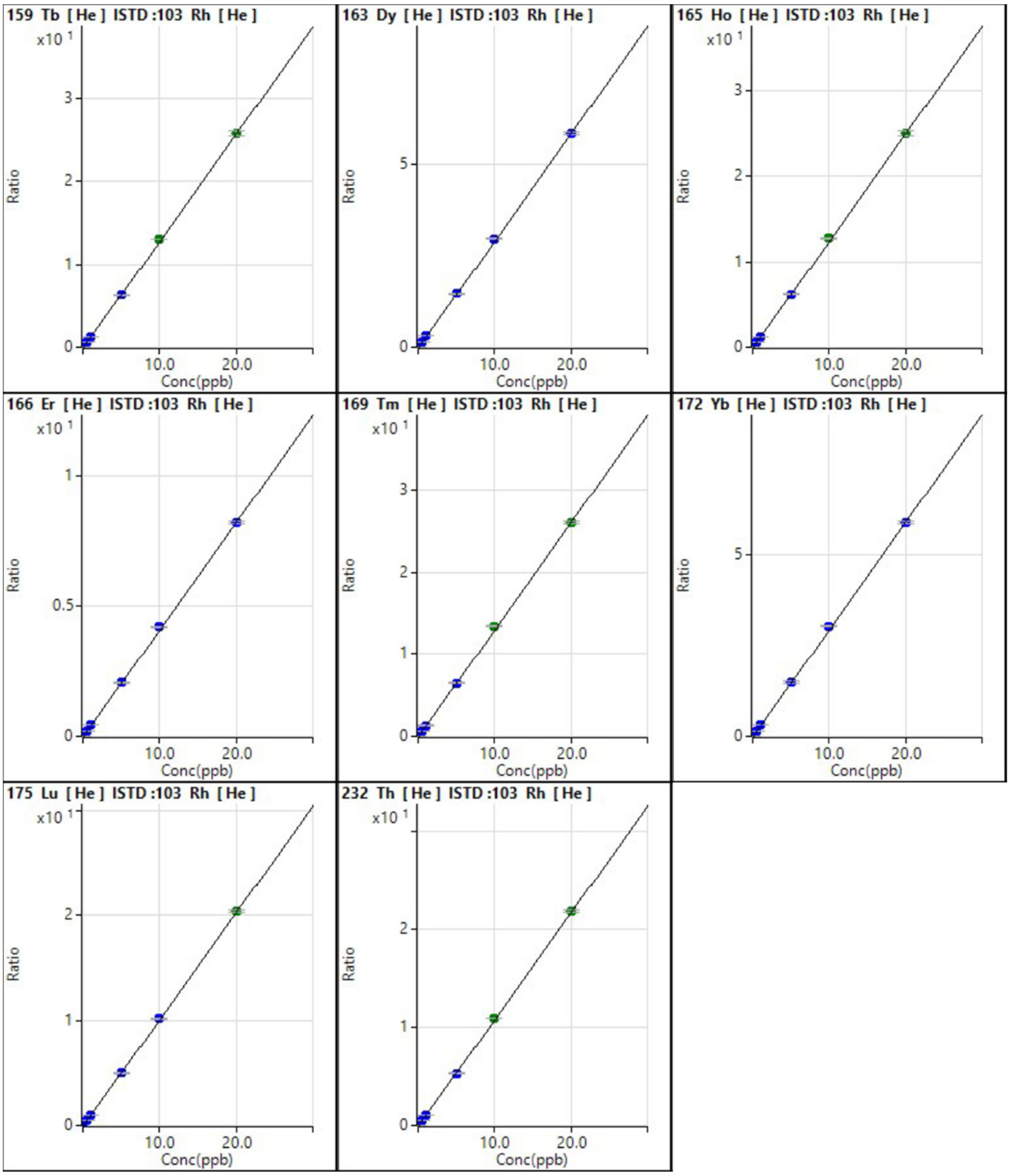

Supplementary Figure S1. ICP-MS calibration performance for the investigated analytes/lanthanides.
